# Bilateral Reduction Mammaplasty as an Oncoplastic Technique for the Management of Early-Stage Breast Cancer in Women with Macromastia

**Published:** 2016-01-14

**Authors:** Russell E. Ettinger, Shailesh Agarwal, Paul H. Izenberg, Richard J. Beil, Daniel G. Sherick

**Affiliations:** ^a^Section of Plastic Surgery and Reconstructive Surgery, Department of Surgery, University of Michigan Medical Center, Ann Arbor; ^b^Center for Plastic & Reconstructive Surgery, St Joseph Mercy Hospital, Ypsilanti, Mich

**Keywords:** breast reconstruction, oncoplastic, reduction mammaplasty, breast cancer, lumpectomy

## Abstract

**Objective**: Lumpectomy may result in contour deformities or breast asymmetry in women with breast cancer and macromastia. This study investigates the use of bilateral reduction mammaplasty, with the tumor and margins included within the reduction specimen. **Methods**: Twenty-four patients who underwent lumpectomy with immediate bilateral reduction mammaplasty for unilateral breast cancer were included. Patient medical records were reviewed for demographic, oncological, and surgical characteristics. **Results**: Mean patient age was 57 years, and mean body mass index was 32.2 kg/m^2^. Mean tumor size was 1.7 cm. All tumor margins were free of neoplastic involvement. No difference was noted between the ipsilateral and contralateral resection weights (*P* = .81). Adjuvant radiation therapy was delivered to 21 patients (88%). There were no significant differences in postoperative total (*P* = .36), major (*P* = .44), or minor (*P* = .71) complications between the tumor and nontumor sides. Only 1 patient required additional revision surgery following the initial lumpectomy with bilateral reduction mammaplasty. **Conclusion**: Lumpectomy with bilateral reduction mammaplasty did not compromise surgical margins. Lumpectomy with bilateral reduction mammaplasty may allow for adequate surgical treatment of breast cancer while avoiding significant breast asymmetry in women with macromastia.

For women with a diagnosis of early-stage breast cancer, breast conservation therapy (BCT)—defined as lumpectomy, followed by adjuvant radiation therapy—is the preferred treatment method.^1^ As the treatment of breast cancer has evolved, the guidelines for BCT have become more inclusive of larger tumors, more advanced disease, and anatomical tumor locations that were previously contraindications for BCT.^2^ Compared with other women, treatment with lumpectomy in women with macromastia can result in greater degrees of breast asymmetry, contour irregularities, and poor aesthetic outcomes that often require additional reconstructive procedures to correct. Furthermore, it is difficult to achieve radiation dose homogeneity in women with large, pendulous breasts, yielding higher rates of radiation fibrosis, chronic pain, and radiation skin changes that further exacerbate any preexisting breast asymmetry and deformity.^3^^-^6

Given the factors confounding lumpectomy outcomes in women with macromastia, the use of lumpectomy and immediate bilateral reduction mammaplasty has been proposed as an oncoplastic method to traditional BCT.^3^^,^^6^^-^12 In this coordinated approach, the tumor resection occurs within the expected glandular resection of the reduction mammaplasty. Following tumor resection, the reduction mammaplasty is carried out on the ipsilateral breast and the unaffected, contralateral breast.

The utilization of lumpectomy and immediate bilateral reduction mammaplasty has been slow to gain acceptance due to concerns regarding oncological safety and the potential for postoperative complications. Spear et al^7^ and Losken et al^11^^,^^13^ have previously shown that this technique is safe and feasible at a large academic center and that it does not negatively affect radiation delivery. However, there remains a relative paucity of data describing the oncoplastic reduction technique when compared with the amount of surgical literature surrounding traditional techniques such as mastectomy or lumpectomy. The need for more studies describing oncoplastic surgery following lumpectomy is underscored by the fact that more women undergo lumpectomy for breast cancer treatment than undergo mastectomy. Finally, most of the studies describing oncoplastic surgery have been based on the experiences of large academic institutions, thereby limiting applicability to smaller or “community” institutions.^4^^,^^7^^,^^8^^,^^11^

In this study, we present our institution's experience with lumpectomy and immediate bilateral reduction mammaplasty for the treatment of breast cancer in women with macromastia.

## METHODS

### Data analysis

An institutional review board–approved (HUM: R-12-1455) retrospective medical record review was conducted on all patients undergoing lumpectomy and immediate bilateral reduction mammaplasty. Patients were identified from the senior surgeons’ (P.H.I., R.J.B., D.G.S.) breast reconstruction practices between 1995 and 2012. All patients were treated at St Joseph Mercy Hospital in Ann Arbor, Mich. St Joseph Mercy hospital is a 537-bed community hospital that serves the greater southeastern Michigan region. Standard criteria were utilized by the referring surgical oncologists in selecting patients for BCT. All patients were initially referred for separate discussions regarding breast oncology management and breast reconstruction following a biopsy-proven primary diagnosis of breast cancer. Only patients with breast cancer localized to a single breast quadrant or central region of the breast as determined on preoperative mammographic imaging were included. Patients with multicentric disease, bilateral breast cancer, or recurrent breast cancer or patients with a prior lumpectomy presenting for *delayed* reduction mammaplasty or mastopexy were excluded. Medical record review of patients meeting inclusion and exclusion criteria was completed evaluating patient demographics, medical comorbidities, tumor size, tumor histology, nodal status, tumor location, skin resection pattern, pedicle type, glandular resection weights, intraoperative alterations to the predetermined reconstructive plan, and perioperative complication rates. Intraoperative breast resection weights from the ipsilateral and contralateral breasts were compared using the Student pairwise *t* test. Perioperative complication rates (total, major/operative, and minor/nonoperative) were analyzed using the χ^2^ test.

### Surgical method

All patients underwent coordinated preoperative planning by the surgical oncologist and senior plastic surgery staff. In all cases, a keyhole-pattern skin resection was marked preoperatively by the senior reconstructive surgeon (P.H.I., RJ.B, or D.G.S.). The tumor resection was first performed by the surgical oncologist through a keyhole-pattern skin marking. Patients undergoing sentinel node biopsy at the time of lumpectomy and reduction mammaplasty received preoperative injection of a radionucleotide tracer and intraoperative injection of isosulfan blue dye. Sentinel lymph node biopsy was carried out following tumor resection. Frozen sections were utilized following tumor resection to verify margin-free status of the primary specimen. After the completion of the cancer resection, the reconstructive surgery team completed the reduction mammaplasty of the ipsilateral breast.

Pedicle type for the ipsilateral breast was selected on the basis of tumor size and location such that the glandular resection would coincide with the tumor. The glandular resection was dictated by the planned area of tumor resection, not vice versa. Pedicle type on the contralateral breast was selected on the basis of plastic surgeon preference and degree of preoperative breast ptosis. Pedicle types included inferior (*n* = 12), superomedial (*n* = 7), central (*n* = 1), bipedicled (*n* = 1), or medial (*n* = 3) for the ipsilateral breast and inferior (*n* = 15), superomedial (*n* = 7), central (*n* = 1), or medial (*n* = 1) for the contralateral breast. Modifications to the glandular resection and pedicle orientation were performed as needed on the basis of the size and location of the tumor resection to prevent compromise of the skin flaps or nipple. All additional glandular tissue removed from the ipsilateral breast was oriented and sent for pathological evaluation. As a final step, the contralateral reduction mammaplasty was completed in the standard fashion and both breasts were evaluated for symmetry on the operating table.

## RESULTS

### Patient characteristics

Twenty-four patients underwent lumpectomy and immediate bilateral reduction mammaplasty for the treatment of primary breast cancer. The mean age at the time of surgery was 57 (SD = 9.6) years. Preoperative breast cup size ranged from C to G as determined on preoperative assessment. The mean body mass index of the group was 32.2 (SD = 6.4) kg/m^2^. No patients were carriers of the *BRCA* gene mutation (Table 1).

### Ipsilateral breast findings

**Figure 1:**
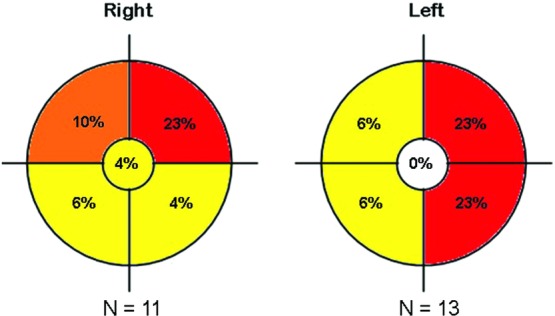
Tumor location based on laterality and breast quadrant.

On the basis of preoperative mammographic imaging, there were 13 tumors of the right breast and 11 of the left (Fig 1). Tumor size ranged from 0.1 to 4.1 cm; all specimens were confirmed to have uninvolved margins on intraoperative frozen sections. Complete tumor resection was confirmed on permanent pathology in all cases—no patients required reoperation for oncological management. Tumor histology included ductal carcinoma in situ (10/24), invasive ductal carcinoma (12/24), and invasive lobular carcinoma (2/24) (Table 1).

### Bilateral reduction mammaplasty approach

A keyhole-pattern skin resection was used for all ipsilateral breasts (24/24). Pedicle type was selected such that the glandular resection would include the tumor resection. An inferior pedicle was utilized most commonly (12/24), followed by superomedial (7/24), medial (3/24), central (1/24), or bipedicled (1/24). Pedicle type for the contralateral breast was based on surgeon discretion. Inferior pedicle was most commonly used (15/24), followed by superomedial (7/24), medial (1/24), or central (1/24).

**Figure 2:**
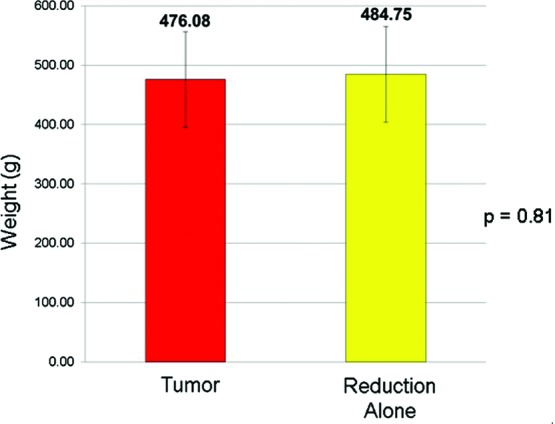
Mean glandular resection weights for the tumor-involved breast and the breast undergoing reduction alone.

All specimens from the breast undergoing reduction alone underwent histological examination, which revealed 2 cases (8%) of previously undiagnosed lobular carcinoma in situ. Mass of glandular resection from ipsilateral breast ranged from 80 to 1403 g (mean = 476 g) and 60 to 1325 g (mean = 485 g) in the breast undergoing reduction alone (Fig 2). We found no statistically significant difference in the resection weights between the ipsilateral and contralateral breasts upon pairwise comparison (*P* = .81).

### Postoperative findings

**Figure 3:**
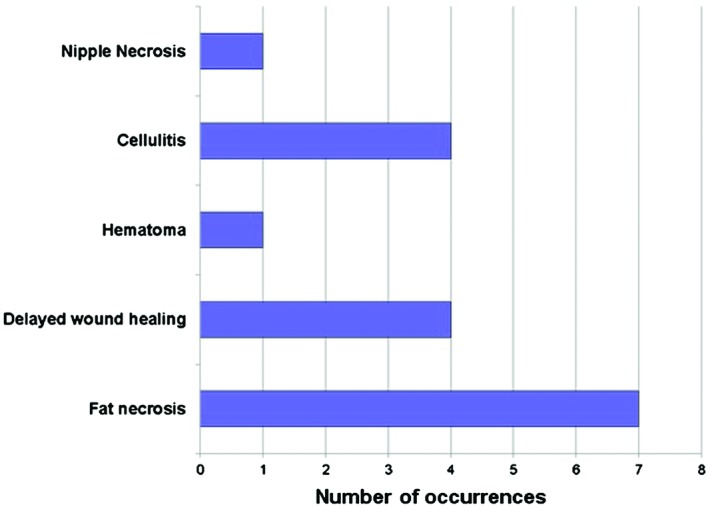
Frequency of postoperative complications.

**Figure 4:**
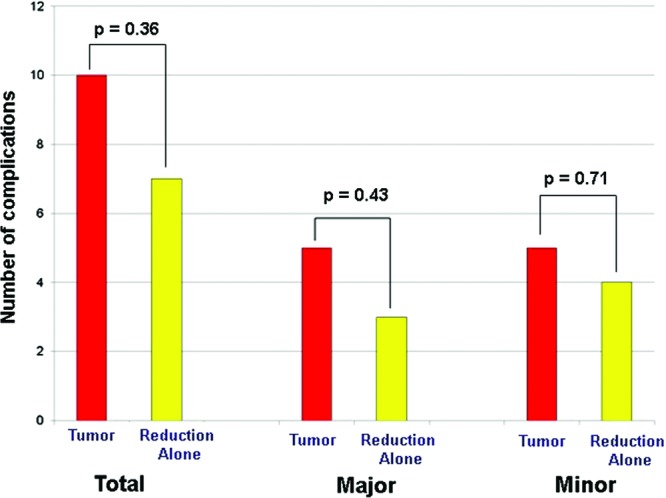
Complication rates by category for the tumor-involved breast and the breast undergoing reduction alone.

Postoperative complications including hematoma, partial nipple necrosis, seroma, fat necrosis, and delayed wound healing were recorded (Fig 3). Complications were dichotomized into major complications, which required operative intervention, and minor complications, which were managed without surgical intervention. There was no statistically significant difference in total, major, or minor complications between the ipsilateral and contralateral breasts (Fig 4). Adjuvant radiation therapy was utilized in 21 of 24 patients. Of the 3 patients who did not receive adjuvant radiation therapy, 2 patients had a low-risk oncotype and received hormonal therapy alone and 1 patient deferred adjuvant treatment despite the recommendations from the treating surgical oncologist. Of the 21 patients who received radiation therapy, 8 patients developed observable breast changes related to radiation therapy as assessed by the treating senior plastic surgeon. None of these patients required operative intervention to address any secondary breast deformity related to radiation therapy. One patient required reoperation due to postoperative asymmetry. No patients experienced a delay in receiving adjuvant radiation or chemotherapy after undergoing lumpectomy and immediate bilateral reduction mammaplasty. One patient required an additional revision procedure to improve symmetry between both breasts.

## DISCUSSION

In this study, we demonstrate that lumpectomy with immediate bilateral breast reduction can be safely performed in patients with macromastia to address a primary breast cancer. No patients had positive margins on final pathology or required re-resection. In addition, the combination of lumpectomy with bilateral breast reduction did not alter the timing or course of radiation therapy or chemotherapy as part of the oncological management. Patients did not experience increased postoperative complications of the ipsilateral breast when compared with the contralateral breast. We noted similar resection volumes of the ipsilateral and contralateral breasts intraoperatively upon pairwise analysis, suggesting that tumor resection did not significantly alter the reduction mammaplasty strategy.

The use of BCT in women with macromastia may lead to suboptimal results secondary to breast asymmetry, poor aesthetic outcomes, and difficulty with adjuvant radiation dose delivery to large breast size.^3^^,^^6^^,^^7^^,^^9^ In the absence of a collaborative approach to surgical planning, patients with macromastia may be offered mastectomy instead. Patients may then undergo reconstruction with additional revision procedures of the ipsilateral and/or contralateral breasts including reduction mammaplasty. The decision to offer patients oncoplastic surgery requires a collaborative approach between breast surgeons and plastic surgeons during the initial discussions regarding surgical management to identify appropriate candidates and inform patients about coordinated techniques for the management of breast cancer.^14^ The need for close coordination between surgical teams also extends into the perioperative and intraoperative periods for the planning of surgical incisions, expected tumor resection, and intraoperative modification technique to ensure optimal outcomes for patients. Although mastectomy with immediate breast reconstruction has certainly increased intraoperative collaboration between surgical oncologists and plastic surgeons, these 2 remain separate entities, both clinically and operatively. However, oncoplastic surgery should be viewed not as 2 separate phases performed by 2 separate surgeons but rather a continuous process designed and executed by a single team.

Spear et al^7^ and Losken et al^11^ have previously demonstrated that lumpectomy with bilateral breast reduction can be safely performed at a large academic institution.^10^ Recently, Egro et al^15^ have demonstrated that patients who undergo reduction mammaplasty at the time of lumpectomy experience fewer complications and obtain improved symmetry when compared with patients who undergo a delayed procedure. It should be noted, however, that 10% to 15% of patients with breast cancer, and an even smaller proportion with early-stage breast cancer, are treated at academic centers.^16^ Therefore, there is a need to demonstrate that oncoplastic surgery can effectively be performed at community hospitals providing care to a significant proportion of patients with breast cancer. Here, we describe the experience of 3 reconstructive surgeons at a 537-bed community hospital. Although this institution does have a loose affiliation with a larger academic medical center, it remains a separate institution with separate staff surgeons. The collaborative approach between the breast surgeons and reconstructive surgeons serves as a model that can be replicated at community hospitals, with results consistent with those reported by others.

Kronowitz et al^8^ have similarly demonstrated lower complication rates associated with immediate reconstruction of partial mastectomy defects. However, they do not advocate simultaneous reduction of the contralateral breast due to concern for changes in ipsilateral breast appearance after radiation therapy.^17^ In our experience, patients were generally satisfied after a single procedure to address both breasts. Only 1 patient sought further revision to obtain improved symmetry at a separate procedure. Typically, patients with macromastia who undergo mastectomy may require tissue expander reconstruction, followed by implant placement, and subsequent revision surgery to improve the symmetry between the larger contralateral breast and the reconstructed breast—this would yield at least 3 surgical procedures in addition to the initial mastectomy. By performing reduction mammaplasty at the time of lumpectomy, we are able to avoid multiple procedures required for traditional reconstruction and subsequent revision procedures. Consistent with our results, Imahiyerobo et al^4^ have shown that oncoplastic reduction does not result in a higher rate of intraoperative complications than that by traditional breast reduction for symptomatic macromastia.

By planning the tumor resection to coincide with the glandular component of the reduction mammaplasty specimen, additional breast tissue can be obtained, allowing for even wider margins than with a traditional lumpectomy. In our series, all patients (24/24) achieved tumor-free margins based on frozen sections and final permanent histological evaluation. Use of frozen section has allowed us to proceed with immediate reconstruction, confident that there will be a low risk for positive margins. During preoperative consultation, all patients were informed that they could require reexcision lumpectomy or a completion mastectomy if final pathological margins were positive, however. Chang et al^3^ have previously reported on the use of lumpectomy with bilateral reduction mammaplasty for patients with unilateral breast cancer, noting that 1 patient did require completion mastectomy due to positive margins on final pathology. However, in their experience, the excised tumor from the ipsilateral breast underwent only gross examination intraoperatively, did not undergo intraoperative frozen section evaluations, and was excised en bloc with the breast reduction specimen. In our reported experience, the tumor was resected separately from the breast reduction specimen and, all resected tumor specimens underwent intraoperative frozen section evaluations.

From an aesthetic standpoint, patients benefit by achieving symmetrically smaller breasts during a single oncoplastic procedure. Although symmetry is determined by what remains, not by what is resected, the fact that the resection weights of the ipsilateral and contralateral breasts were similar in each patient suggests that the surgeon was comfortable obtaining symmetry in the patient. Furthermore, patients at follow-up were satisfied with their operative outcome based on discussion between the patient and the treating plastic surgeon and only 1 patient required reoperation to improve symmetry. The reduction of breast size in patients with macromastia may also improve upon the known functional sequelae of macromastia including neck pain, shoulder pain and grooving, and poorly fitting clothing. In addition, adjuvant radiation therapy is facilitated by smaller breast size, with the potential for improved radiation dose homogeneity in the ipsilateral breast.^18^

Two patients (8%) were found to have a synchronous lobular carcinoma in situ of the contralateral breast, which was contained within the reduction mammaplasty resection. Neither patient required further intervention for the previously undiagnosed lobular carcinoma in situ. Chang et al^3^ found a similar rate (5.5%) of synchronous tumors in women undergoing concurrent lumpectomy with reduction mammaplasty. Although this may provide further intervention to examine additional breast tissue, we do not advocate contralateral breast reduction as a method for contralateral breast screening.

Fat necrosis was the most common complication, followed by delayed wound healing and cellulitis. Nipple necrosis and hematoma were less frequently occurring once each. We report a relatively high total complication rate of 54.2%, which is likely due to our inclusion of minor complications such as all forms of delayed wound healing, fat necrosis, and cellulitis, which did not require operative intervention. Upon pairwise comparison of the ipsilateral and contralateral breasts for complications, we found no statistically significant difference in the total (*P* = .36), major (*P* = .43), or minor complications (*P* = .71) between the ipsilateral and contralateral breasts. This suggests that tumor resection at the time of reduction does not increase the complication risk. However, we note that the power of our study is limited, as we include only 24 patients. Nonetheless, the ability to combine cancer resection with immediate reconstruction may additionally mitigate the morbidity associated with having multiple operations.

In our series, 21 of 24 patients (88%) underwent adjuvant radiation therapy after combined lumpectomy and reduction mammaplasty. Eight patients were noted to have significant changes in their breast skin and contour following radiation therapy, but none required operative intervention to address these changes. Postradiation sequelae have been demonstrated in previous studies,^3^^,^^7^ occurring with a frequency of up to 53%.^10^ However, no patients in previous series required further operative intervention related to radiation changes, which is consistent with our experience. No patients experienced delays in time to radiation or chemotherapy in our study.

Our study has the usual limitations associated with a retrospective review at a single institution. Although we demonstrate no significant differences between the contralateral and ipsilateral breasts in terms of complication rates, we note that this may be due to a small sample size. However, we demonstrate that oncoplastic surgery in the form of lumpectomy with immediate bilateral reduction is an oncologically safe technique in women with macromastia. No women had positive margins or required reexcision in our experience. Only 1 patient required additional revision surgery, in contrast to the number of operations required for patients undergoing traditional reconstruction following mastectomy. This technique is feasible not only at large academic institutions, as has been previously demonstrated, but also at hospitals in the community setting. As patient care and even legislation demand a collaborative approach between plastic surgeons and surgical oncologists, this technique deserves further evaluation to encourage broader implementation.

## Figures and Tables

**Table 1:** Demographic and clinical variables*

Variable		Range	SD
Mean age, y	57.0	40–71	9.6
Mean BMI, kg/m^2^	32.3	23–47	6.6
Comorbidities			
Hypertension	10		
Dyslipidemia	3		
Diabetes	2		
Depression	3		
Hypothyroidism	2		
Headache	4		
Smoking			
Active	0		
Former	11		
Nonsmoker	13		
*BRCA* positivity	0/24		
Tumor laterality			
Right	13		
Left	11		
Tumor size, cm	1.7	0.1–4.1	1.5
Tumor type			
DCIS	10		
Invasive ductal	12		
Invasive lobular	2		
Adjuvant radiation	21/24		

*BMI indicates body mass index; DCIS, ductal carcinoma in situ.
